# Towards a Dimensional, Multifactorial, and Integrative Approach to Procrastination in Everyday Life: An Illustration through Interviews

**DOI:** 10.5334/pb.1115

**Published:** 2022-04-18

**Authors:** Marie My Lien Rebetez, Catherine Barsics, Timothé Montisci, Lucien Rochat

**Affiliations:** 1 Cognitive Psychopathology and Neuropsychology Unit, University of Geneva, Geneva, CH; 2 Department of Psychiatry, Geneva University Hospitals, Geneva, CH

**Keywords:** procrastination, everyday life, adaptive and maladaptive procrastination, interviews, qualitative content analysis, dimensional approach

## Abstract

Procrastination is a widespread phenomenon that has been extensively studied but about which a clear and integrated picture is still lacking, as reflected in the multiplicity and diversity of its definitions, causes and consequences. In addition, its examination in everyday life has been somewhat overlooked. The aim of this paper is to further the understanding of procrastination, first by providing an overview of its various definitions, causes, and consequences. Using a qualitative approach, we then provide an in-depth descriptive account of procrastination episodes retrospectively reported by six participants from the general population in diverse situations of their daily life, focusing in particular on the definitions, causes, and consequences of procrastination behaviours. Finally, this descriptive account of procrastination is discussed in terms of a dimensional, multifactorial, and integrative approach.

## Introduction

Procrastination, or “putting off until a later time,” is a widespread phenomenon that has been extensively studied (see Steel, 2007). However, the exact nature of this phenomenon is still debated (Wilson & Nguyen, 2012), as reflected in the wide variety of definitions and the multiplicity and heterogeneity of causes and consequences that have previously been postulated. In addition, most studies have focused on academic procrastination (i.e., delay of study-related activities in a student population) while only a few studies have examined procrastination in everyday life (Klingsieck, 2013a).

In this context, the purpose of this paper was to further the understanding of procrastination in everyday life. First, we provide a comprehensive overview of the literature on the definitions, causes, and consequences of procrastination. Second, using a qualitative approach, we provide an in-depth descriptive account of this phenomenon from the retrospectively reports on dilatory behaviours in various daily life domains of six participants from the general population. This is examined, on the one hand, through its definitions and, on the other, through its causes and consequences. This descriptive account is finally discussed in light of a dimensional, multifactorial, and integrative approach to procrastination.

### Definitions of procrastination

As stated by Ferrari, Johnson, and McCown (1995, p. 5), *“a major difficulty in studying, understanding, and treating procrastination may involve variations in its subjective definitions.”* Indeed, there are almost as many definitions of procrastination as there are researchers writing about it. The notion of “delay,” however, appears to be a common feature to the various definitions. According to Ferrari et al. (1995), this delay has to be “voluntary” (i.e., resulting from a deliberate, purposeful choice). Other authors (e.g., Beswick & Mann, 1994) add the notion of “intentionality” (i.e., the start or the completion of the action is intended). Yet other contributors (e.g., Silver & Sabini, 1981) highlight the notion of “irrationality” (i.e., delaying is illogical, unnecessary, and/or occurs despite awareness of potential negative consequences). Combining these different notions, Steel (2007, p. 66) proposed the following definition: *“to procrastinate is to voluntarily delay an intended course of action despite expecting to be worse off for the delay.”*

However, as Steel (2007) himself points out, procrastination is occasionally used in a positive sense. Chu and Choi (2005) thus introduced the term *active procrastination* to refer to an adaptive form of delay associated with four aspects: preference for time pressure, intentional decision to procrastinate, ability to meet deadlines, and satisfaction with outcome. It is worth noting, though, that several authors questioned this conception (e.g., Chowdhury & Pychyl, 2018; Corkin, Yu, & Lindt, 2011). More specifically, Chowdhury and Pychyl (2018) demonstrated that active procrastination was not related to a self-report measure of procrastination behaviour and was negatively related to other procrastination measures (i.e., procrastination intensity, general procrastination), whereas it showed positive relations with purposeful delay (delaying tasks deliberately according to the external demands) and arousal delay (delaying tasks to feel the time pressure). Hence, the authors argued that active procrastination is a deliberate delay that is purposeful, but cannot be considered procrastination.

Finally, with the aim of providing a clear distinction between procrastination – hence conceived as maladaptive – and adaptive forms of delay, Klingsieck (2013a, p. 26) proposed that procrastination is *“the voluntary delay of an intended and necessary and/or [personally] important activity, despite expecting potential negative consequences that outweigh the positive consequences of the delay.”* She also identified the seven following aspects as being constitutive of procrastination definitions: (1) *“an overt or covert act is delayed,”* (2) *“the start or the completion of this act is intended,”* (3) *“the act is necessary or of personal importance,”* (4) *“the delay is voluntary and not imposed on oneself by external matters,”* (5) *“the delay is unnecessary or irrational,”* (6) *“the delay is achieved despite being aware of its potential negative consequences,”* and (7) *“the delay is accompanied by subjective discomfort or other negative consequences.”*

### Causes and consequences of procrastination

A multiplicity of causes and consequences of procrastination adds up to the diversity of its definitions. As illustrated by Steel (2007) in a meta-analytic and theoretical review of procrastination based on 691 correlations, a multitude of links between this phenomenon and a wide variety of individual and situational variables has been studied since the late 1970s.

#### Causes

Procrastination has been shown to be closely associated with low conscientiousness (i.e., lack of organization, persistence, control, and motivation in goal-directed behaviour; Schouwenburg & Lay, 1995) and conceptualized as a self-regulatory failure (Steel, 2007). In this conception, procrastinators differ from non-procrastinators in the degree to which they act upon their intentions (Steel, Brothen, & Wambach, 2001). More specifically, this self-regulatory failure has been associated with thought control problems (e.g., distractibility, daydreaming, rumination; Flett, Stainton, Hewitt, Sherry, & Lay, 2012; Harriott, Ferrari, & Dovidio, 1996; Stainton, Lay, & Flett, 2000), a high level of impulsivity (e.g., Dewitte & Schouwenburg, 2002; Gustavson, Miyake, Hewitt, & Friedman, 2014; Rebetez, Rochat, & Van der Linden, 2015), and a strong preference for immediacy (e.g., O’Donoghue & Rabin, 2001; Reuben, Sapienza, & Zingales, 2015). Individual differences in time perspectives have also been demonstrated, indicating that procrastinators are less likely to use a future time orientation to guide their decisions and actions (for a review, see Díaz-Morales & Ferrari, 2015; Sirois, 2014).

Moreover, the self-regulation problems of procrastinators have been considered from the perspective of their emotion regulation strategies: procrastinators prioritize the management of immediate mood over long-term goal pursuit (e.g., voluntary delay of an intended task viewed as aversive to repair the negative mood surrounding the task) due to a disconnection between present and future self (Blouin-Hudon & Pychyl, 2015; Sirois & Pychyl, 2013). Other data underline the role of self-related factors in procrastination such as self-efficacy (e.g., Haycock, McCarthy, & Skay, 1998) and self-esteem (e.g., Ferrari, 1994). More specifically, in delaying the start or completion of a task, procrastinators avoid the risk of failure and the test of their abilities, thereby protecting their (social) self-esteem (Ferrari, 1991). The role of perfectionism in procrastination, which is related to worry about receiving negative evaluations, has also been discussed, but its contribution to procrastination remains debated (for a discussion on that topic, see Pychyl & Flett, 2012).

Findings on the relationships between procrastination and different constructs of motivation (e.g., intrinsic or extrinsic motivation; in other words, motivation resulting from internal drives or from external contingencies) are heterogeneous, showing links with intrinsic and/or extrinsic motivation (e.g., Brownlow & Reasinger, 2000; Lee, 2005; Senécal, Koestner, & Vallerand, 1995; Solomon & Rothblum, 1984), or with amotivation (Lee, 2005; Senécal et al., 1995). Finally, some people may procrastinate for reasons of arousal (e.g., to get the “rush” from completing a task close to the deadline; Ferrari, 1992) or because of the (false) belief that they work better under pressure (Simpson & Pychyl, 2009).

Further adding to the challenges in understanding the nature of procrastination, Choi and Moran (2009, p. 209) argued that, in contrast to the classic view of procrastination as a self-regulatory failure, *“active procrastination is driven by a strong self-regulatory process.”* Unlike “passive procrastinators” (i.e., procrastinators in the traditional sense), who do not intend to procrastinate but often end up doing so because of difficulties in acting on their intentions, “active procrastinators” deliberately decide to delay (Choi & Moran, 2009; Chu & Choi, 2005). In addition, active procrastination has been associated with the capacity to flexibly manage time and to motivate oneself under time pressure, as well as self-efficacy beliefs, adaptive stress-coping strategies, emotional stability, extraversion, and a preference for multitasking (Choi & Moran, 2009; Chu & Choi, 2005). It should be borne in mind, however, that according to several authors (e.g., Chowdhury & Pychyl, 2018; Corkin et al, 2011), active procrastination cannot be labelled as procrastination inasmuch as it is crucially considered a self-regulatory failure. In this respect, Chowdhury and Pychyl (2018) showed that the pattern of relations found for active procrastination (as Chu & Choi, 2005) was similar to those found for purposeful delay but were different from the pattern observed with “traditional” procrastination (i.e., representative of self-regulatory failure).

Whether delay is considered adaptive or maladaptive, the most frequently reported cause of procrastination is task aversiveness (Steel, 2007). Researchers have observed that the delayed task/action, although perceived as important (e.g., Lay, 1986; Milgram, 1991), is considered unattractive, boring, stressful, difficult, highly effortful, or unclear (e.g., Blunt & Pychyl, 2000; Ferrari, Mason, & Hammer, 2006; Ferrari & Scher, 2000; Pychyl, Lee, Thibodeau, & Blunt, 2000; Schraw, Wadkins, & Olafson, 2007). The perceived aversiveness of a task or an action may, however, vary according to the context or as a function of time; for example, task attractiveness may diminish when an individual is faced with another task providing a more immediate gratification, but may increase as the deadline approaches (Schouwenburg & Groenewoud, 2001). In addition, Klingsieck (2013b), in examining the frequency of procrastination in different life domains (academic/work, everyday routines/obligations, health, leisure, family/partnership, social contacts), demonstrated that procrastination was domain specific. Indeed, a confirmatory factor analysis performed on procrastination frequency in each of the six aforementioned domains indicated that a six-factor model (conceiving procrastination as domain specific) yielded a better fit than did a one-factor model (conceiving procrastination as domain general). Furthermore, significant differences in procrastination frequency across domains were found, with the highest frequency in academic/work, followed by everyday routines/obligations and health, social contacts and family/partnership, and finally leisure.

#### Consequences

The list of negative consequences of procrastination is long, having adverse impacts on academic performance (e.g., Tice & Baumeister, 1997), career and financial success (e.g., Mehrabian, 2000), or mental health (e.g., lower level of well-being and higher level of distress, feelings of shame, or guilt; Blunt & Pychyl, 2005; Fee & Tangney, 2000; Sirois & Tosti, 2012; Stead, Shanahan, & Neufeld, 2010). Research also reveals physical health repercussions of procrastination via increased stress and fewer behaviours related to health prevention, maintenance, and/or enhancement (such as medical and dental check-ups, diet, or exercise; Sirois, 2007; Sirois, Melia-Gordon, & Pychyl, 2003).

However, procrastination, or at least purposeful delay, does not always appear to lead to negative consequences and might be associated with positive and constructive outcomes. As previously noted, active procrastination has been associated with the ability to meet deadlines and satisfaction with the outcome (Chu & Choi, 2005). Chu and Choi (2005) additionally mentioned associations with low levels of stress and depression and high levels of life satisfaction and performance. Still, other positive consequences are the maximization of learning in a minimal amount of time, the achievement of a state of flow (i.e., state of total involvement in an activity), and the enhancement of motivation (Schraw et al., 2007).

## The present study

Thus, the existing literature has demonstrated that procrastination is a complex phenomenon associated with a host of causes (individual and situational) and consequences (positive and negative), which is still not fully understood. In particular, the numerous and sometimes contrasting definitions of procrastination have made this phenomenon difficult to apprehend. As a result, *“most of the research on procrastination is not driven by a commonly shared theory”* (van Eerde, 2003, p. 1). Indeed, research on the matter falls within different perspectives (e.g., perspectives that understand procrastination as a personality trait versus those that focus on context-related variables; Ferrari, 2010; Klingsieck, 2013b) or relates to specific psychological theories, each focusing on particular aspects of procrastination, such as self-determination theory (Deci & Ryan, 1985; e.g., Senécal, Julien, & Guay, 2003), self-efficacy theory (Bandura, 1977; e.g., Klassen, Krawchuk, & Rajani, 2008), appraisal-anxiety-avoidance theory (Lazarus & Folkman, 1984; Milgram & Tenne, 2000), or action control theory (Kuhl, 1984; e.g., Blunt & Pychyl, 2005). Furthermore, research on procrastination has mainly applied quantitative methodologies in which data have been collected through self-report questionnaires.

In contrast, few authors have used a qualitative approach, but their studies have provided new insight into procrastination through the examination of academic procrastination according to students’ subjective experiences (Grunschel, Patrzek, & Fries, 2013; Klingsieck, Grund, Schmid, & Fries, 2013; Lindblom-Ylänne, Saariaho-Räsänen, Inkinen, Haarala-Muhonen, & Hailikari, 2015; Schraw et al., 2007). Schraw et al. (2007) interviewed about 60 students regarding their own experiences of academic procrastination, from which these investigators built a process model of procrastination. This model accounted for the antecedents of procrastination (e.g., personal interest in a task, task characteristics, teacher expectations), the phenomenon of procrastination itself (covering both adaptive and maladaptive aspects), context and conditions (unclear directions, deadlines, lack of incentives), cognitive and affective strategies used to cope with procrastination (e.g., identifying clear goals, positive reframing of procrastination behaviour), and consequences (both on quality of life and quality of work). Interestingly, the authors noted that the majority of interviewed students viewed procrastination in a positive way (e.g., increasing the likelihood of achieving a state of flow or maximizing learning in a minimal amount of time). The authors also observed that the students linked procrastination to a wide variety of factors (although none of these factors taken in isolation seemed to cause procrastination), and identified both positive and negative consequences on quality of life (although little impact on quality of work was reported). This qualitative study thus highlighted significant aspects of procrastination, in particular adaptive aspects, from the participants’ point of view. However, the study is limited in that it focused only on procrastination in the academic domain.

In this context, we aimed to expand the understanding of procrastination – defined as putting off until a later time – by providing comprehensive descriptions of procrastination in the general population with no restriction on life domains. More specifically, we reported an in-depth descriptive account of the participants’ views on the characteristics and manifestations of procrastination across various domains of their daily life, allowing to (a) examine whether the six core aspects of procrastination that Klingsieck (2013a) collected from previous definitions were fulfilled by the participants’ descriptions, (b) develop a systematic categorisation of causes (individual and situational) and consequences (positive and negative) expressed by the participants, and further explore how these causes and consequences combine in each participant and situation.

## Methods

### Data collection

Face-to-face semi-structured interviews (Schilling, 2006; Tesch, 1990) were conducted with six volunteers from the community (three females and three males between the ages of 26 and 42) who were recruited through personal contacts but who did not personally know the people conducting the interviews and the analyses; they received no compensation for their participation. The inclusion criterion was being a fluent speaker of French with a mean score on a measure of trait procrastination (French adaptation of the Pure Procrastination Scale, PPS; Rebetez, Rochat, Gay, & Van der Linden, 2014) that was higher than 2.66 (2.66 corresponding to the mean obtained in the French validation study of the PPS; Rebetez et al., 2014). Participant 3 had the highest score of procrastination (PPS mean score, m = 3.73; percentile rank, PR based on the French validation study of the PPS; Rebetez et al., 2014 = 90), followed by participant 4 (m = 3.64; PR = 87), 6 (m = 3.36; PR = 80), 1 (m = 3.09; PR = 70), 5 (m = 3.00; PR = 65) and 2 (m = 2.73; PR = 54). Thus, the participants’ score on the PPS ranges from medium to elevated. At the beginning of the interview, procrastination was broadly defined by the interviewer as “putting off until a later time.” Participants were then asked to describe three situations in which they procrastinated (from any time period). They were next asked about the reasons and consequences of their procrastination pertaining to each expressed situation. Subsequent questions (adapted from Grunschel et al., 2013) were used to stimulate the depth of participants’ answers (e.g., Which other reasons for procrastination come to your mind? Are there any other consequences, positive or negative?). The interview procedure was approved by the local Ethics Committee and informed consent was obtained from participants before the onset of the interview. All interviews, which lasted on average 46.3 minutes (*SD* = 10.5, range = 34–59), were tape-recorded and fully transcribed.

### Data analysis

Interviews were segmented into idea units (i.e., *“a segment of text that is comprehensible by itself and contains one idea, episode, or piece of information”*; Tesch, 1990, p. 116) and this content was analysed by using qualitative analysis procedures (Schilling, 2006). Qualitative analysis organizes data across distinct categories that can be developed deductively (i.e., theoretically derived from the literature) or inductively (i.e., content based).

In order to examine how constituent parts of procrastination definitions were met by different individuals facing distinct situations, we applied the deductive approach, using Klingsieck’s (2013a) definition as a theoretical basis. The following categories were derived from the criteria of the definition: 1 = an overt or covert act is delayed, 2 = the start or the completion of this act is intended (evidence that the act has been intended, anticipated, or started), 3 = the act is necessary or of personal importance, 4 = the delay is voluntary and not imposed on oneself by external matters, 5 = the delay is irrational (the fifth and sixth criteria of Klingsieck’s definition were combined, in consideration that unnecessary delaying or achieving a delay despite being aware of its potential negative consequences are manifestations of irrationality), and 6 = the delay is accompanied by subjective discomfort or other negative consequences. The content of each interview was reviewed independently by the first and fourth authors, and categorized according to the system described above. Critical idea units were discussed until a consensus had been reached.

For the purpose of obtaining multifaceted descriptions of procrastination from different participants’ perspectives across various domains of daily life, the deductive and inductive approaches were combined. Categories were deductively developed from the current literature on procrastination and inductively from a small set of interviews. The following main categories (subcategories) were initially created: causes (individual and situational) and consequences (negative and positive). Further interviews were coded on the basis of this category system by the first author and reviewed by the fourth author. New categories were added when idea units did not fit into existing categories, categories were split when they were too heterogeneous and combined when they overlapped, and the revised category system was applied to another set of interviews. This procedure was repeated several times until all idea units were classified.

## Results

Overall, the six participants generated 17 different situations (one respondent was able to describe only two situations) in which they had procrastinated. Procrastination was experienced in various life domains, namely, Academic/Work (AW), Everyday routines/Obligations (EO), Health (H), and Social contacts (S).

### Constituent parts of procrastination definitions

**Figure 1:**
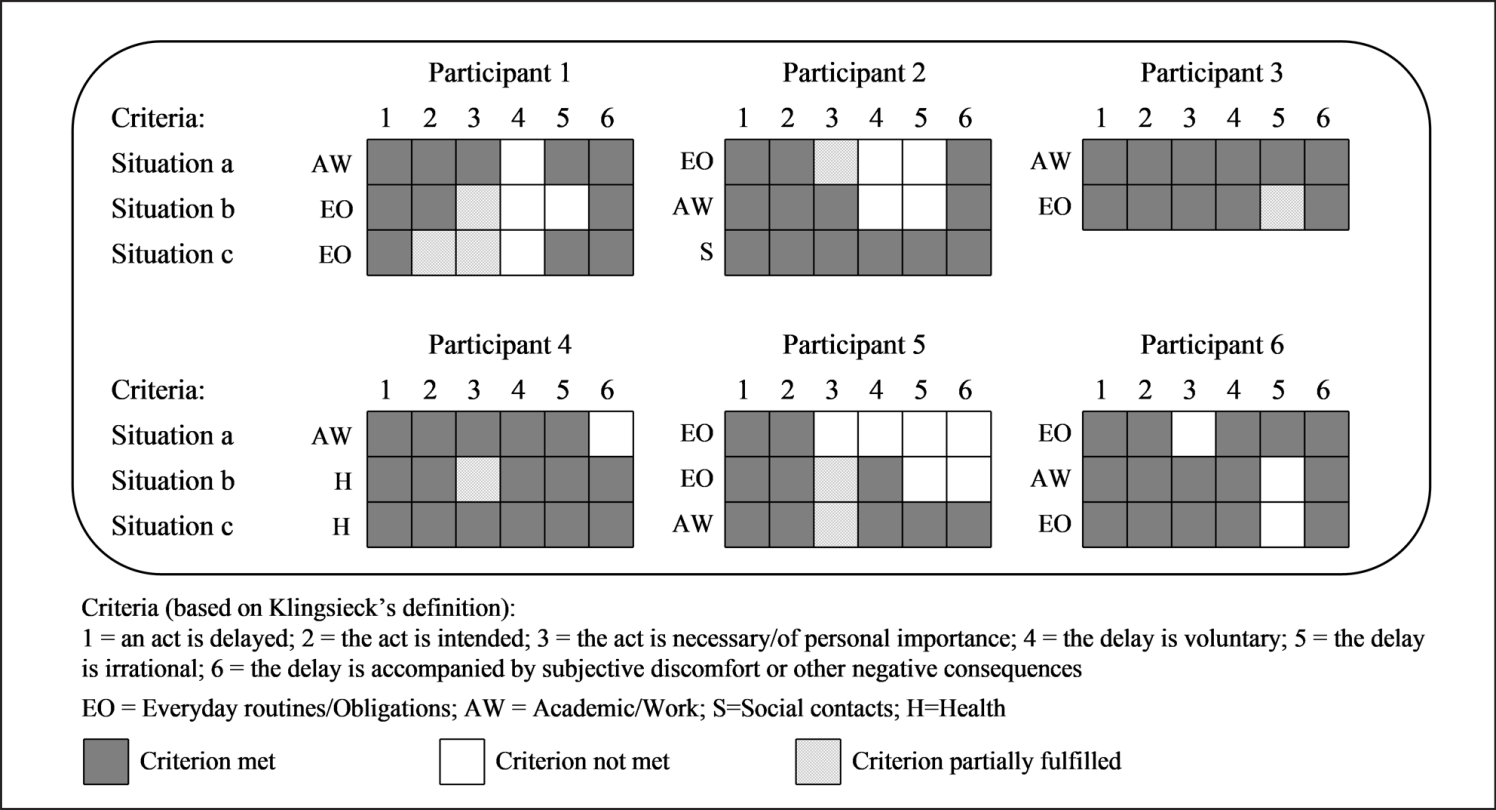
Criteria of procrastination definitions met by the participants according to the situation.

Within the 17 situations described by the six participants, 221 idea units were identified as containing constituent parts of procrastination definitions (see ***Figure 1***).

Only three participants (Participants 2 to 4) in one situation each (situations c: phoning an old friend, a: revising for a continuous assessment test, c: initiating drug therapy, respectively) met all criteria regarding constituent parts of procrastination definitions. Given that participants were asked to describe situations of procrastination defined as “putting off until a later time”, Criterion 1 (i.e., an overt or covert act is delayed) was therefore met in all participants across all situations. However, Criteria 2 to 6 were not met or were only partially fulfilled by many participants and in several situations.

#### Criterion 2: the start or the completion of the act is intended

The intention to start or complete the act was ambivalent in Participant 1 (c: weekly shopping): *“Well, I went shopping one day at noon, but I just bought a few items. And I thought maybe I was not going to do the rest.”*

#### Criterion 3: the act is necessary or of personal importance

For Participants 1 (b: buying foam mats, c: weekly shopping), 2 (a: daily shopping), 4 (b: making a doctor’s appointment), and 5 (b: storing toilet paper rolls, c: calling to receive information about a job), the necessity or personal importance of the act was ambiguous: e.g., *“No, it [storing toilet paper rolls] was not important. But yes, it was important, because I don’t want to live in chaos and disorder.”* For Participants 5 (a: opening mail) and 6 (a: terminating the lease contract of a parking space), the act was not at all necessary or personally important (e.g., *“It was not as if it was important for me”*).

#### Criterion 4: the delay is voluntary and not imposed on oneself by external matters

Criterion 4 was not met by Participants 1 (a: preparing a presentation for a meeting, b: buying foam mats, c: weekly shopping), 2 (a: daily shopping, b: producing a report for a client), and 5 (a: opening mail), meaning that the delay ensued, at least partly, from external constraints: e.g., unfavourable context (*“In my working environment, I am disturbed all the time. I get phone calls, emails… My work is continuously interrupted”*), missing data (*“Some elements necessary to begin work were missing”*), dependence on others for the realization of the act (*“I also worked with other people who were not ready”*), other obligations (*“I had other things to deal with”*), unforeseen events (*“There was a disruptive element, namely, unplanned work sessions”*), and time available (*“It was rather a question of time, because when I came home from work, it was too late to go to the shopping centre”*).

#### Criterion 5: the delay is irrational

Criterion 5 was not met in Participants 1 (b: buying foam mats), 2 (a: daily shopping, b: producing a report for a client), 5 (a: opening mail, b: storing toilet paper rolls), and 6 (b: revising for a written examination, c: booking service for a scooter), meaning that they deliberately and rationally chose to modify their plans: e.g., *“I prefer doing shopping than cleaning. I, however, chose to vacuum because I had not done it for two weeks. Shopping could wait.”* For Participant 3 (b: filling out a tax return), the irrationality of the delay was somewhat ambiguous: *“I did an apprenticeship in the tax services, I know how it works. I planned to postpone it until July but to do it just before leaving on holidays, in order to avoid fines. But it’s silly because it does not take long.”*

#### Criterion 6: the delay is accompanied by subjective discomfort or other negative consequences

Finally, Criterion 6 was not met in Participants 4 (a: completing a project for a competition) and 5 (a: opening mail, b: storing toilet paper rolls): e.g., *“Actually, it [procrastination] had no consequence at all.”*

### Multifaceted descriptions of procrastination

**Figure 2:**
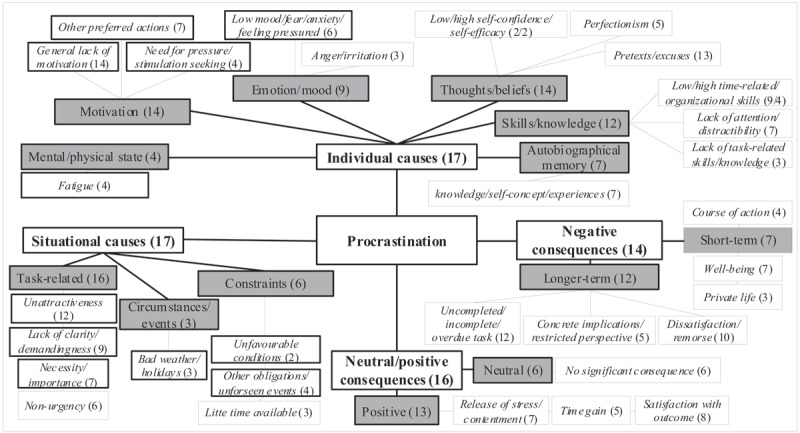
Causes and consequences of procrastination: **main categories**, subcategories, *themes*. *Note*: Numbers represent the number of participants mentioning at least one statement per situation in each category.

Four hundred forty-five idea units were identified as containing causes and consequences of procrastination. Their analysis yielded two category systems: causes (differentiated into two **main categories**, nine subcategories, and 21 *themes*) and consequences (differentiated into two **main categories**, four subcategories, and 10 *themes*; see ***Figure 2***).

#### Causes-category system

Regarding the causes-category system, **individual** and **situational causes** (**main categories**) were distinguished. The **individual causes** were divided into the following subcategories (and *themes*): mental/physical state (comprising one theme referring to participants’ *fatigue*), motivation (including themes ranging from *general lack of motivation* and *other preferred actions* to *need for pressure/stimulation seeking*), emotion/mood (*low mood/fear/anxiety/feeling pressured; anger/irritation*), thoughts/beliefs (both low and high *self-confidence/self-efficacy; perfectionism*; resort to *pretexts/excuses* for postponing), skills/knowledge (both low and high *time-related/organizational skills; lack of attention/distractibility; lack of task-related skills/knowledge*), and autobiographical memory (referring to participants’ autobiographical *knowledge/self-concept* with regard to procrastination or specific *experiences* such as being successful despite procrastinating). The **situational causes** were divided into task-related characteristics (*unattractiveness; lack of clarity/demandingness*; both low and high *necessity/importance; non-urgency*) and circumstances/events subcategories (*bad weather/holidays*). Another subcategory, namely, constraints, referred to conditions not mentioned as causes for procrastinating but as hindering/slowing down the course of action: this subcategory includes *unfavourable conditions* (such as unfavourable context, missing data, or dependence on others for the realization of the task/action), *other obligations/unforeseen events*, and *little time available*.

#### Consequences-category system

With respect to the consequences-category system, **negative** and **neutral/positive consequences** were distinguished (**main categories**). The **negative consequences** were divided into short-term consequences (effect on participants’ *course of action* such as needing to work more intensively, *well-being*, and *private life*) and longer-term consequences subcategories (*uncompleted/incomplete/overdue task; concrete implications/restricted perspective; dissatisfaction/remorse*). The neutral consequences subcategory includes statements clearly describing the *absence of significant consequence*. The positive consequences subcategory was divided into *release of stress/contentment, time gain*, and *satisfaction with outcome*.

#### Examination of idea units for each participant and situation

Further analysis of idea units showed a considerable heterogeneity among causes and consequences according to the participants and situations; see ***Table 1*** below for examples of participants’ descriptions across different situations. Some participants named more individual than situational causes for procrastination (see below, Participants 3 or 6), while others were more balanced (see Participants 1, 2 or 4). Most participants mentioned a general lack of motivation as a cause of procrastination in all situations, but one participant reported no motivational reason at all (see Participant 5). The use of pretexts/excuses for postponing was also mentioned by many participants in several situations. Within the same individual (see Participants 3 or 6), dysfunctional aspects of procrastination (e.g., fear, anxiety, low self-confidence, lack of task-related skills) were more frequently expressed in certain situations (e.g., everyday routines/obligations) than in others (e.g., academic/work), with the latter instead involving functional aspects such as good time-related/organizational skills and high self-efficacy. By contrast, some causes (e.g., fatigue, low time-related/organizational skills, lack of attention/distractibility or perfectionism) were reported regardless of the situation (see Participants 1, 3 or 6). Looking at situational causes, we found that the unattractive nature of the task was usually reported as a reason for procrastinating, while other task characteristics varied across situations (e.g., everyday routines/obligations were associated with little necessity/importance, whereas work was perceived as highly important; see Participant 2). Finally, some participants reported numerous consequences (both negative and positive) in all situations (Participants 1 or 2), whereas other participants identified consequences in certain situations but not in others (Participant 3), or reported few consequences (Participant 5).

**Table 1:** Examples of participants’ descriptions across different situations.


Participant 1, male, 37 years, urban planner; PPS m = 3.09, PR = 70			

**Situations a (AW):** preparing a presentation for a meeting (Criterion 4 not met)	**b (EO):** buying foam mats (Criterion 3 partially fulfilled, Criteria 4–5 not met)		**c (EO):** weekly shopping (Criteria 2–3 partially fulfilled, Criterion 4 not met)

**Individual causes**			

**Mental/physical state: *fatigue*** *(“I was tired”)*	**-**		**-**

**Motivation: *general lack of motivation*** *(“I was not really motivated”)*; ***need for pressure/stimulation seeking*** *(“I only started to work when I felt stressed, under pressure”)*	***general lack of motivation***; ***other preferred actions*** *(“I preferred to do other things; to do things of interest to me”)*		***general lack of motivation***; ***other preferred actions***

**Emotion/mood: -**	***anger/irritation*** (*“Because it got on my nerves”*)		***anger/irritation***

**Thoughts/beliefs:**high ***self-confidence/self-efficacy*** *(“I knew I could do it”)*	***pretexts/excuses*** *(“I always found some excuse, such as I don’t have time, it’s too far away, the weather is not nice”)*		***pretexts/excuses***

**Skills/knowledge:** low ***time-related/organizational skills*** *(“I didn’t realize that it would take so much time”; “I did other things that I could have done afterwards”)*; ***lack of task-relatedskills/knowledge*** *(“I didn’t know how to do it”)*	low ***time-related/organizational skills***		low ***time-related/organizational skills***

**Autobiographical memory: -**	**-**		**-**

**Situational causes**			

**Task-related characteristics: –**	***unattractiveness*** *(“It’s really boring, I hate to go to malls”)*; low ***necessity/importance***;***non-urgency*** *(“It’s not something urgent ; I don’t have a deadline”)*		***unattractiveness***; low ***necessity/importance*** *(“I don’t really need a fully stocked fridge, therefore it’s not that necessary”)*

**Circumstances/events: -**	**-**		**-**

**Constraints: *unfavourable conditions*** *(“Since I work in an open space, I am constantly interrupted when I work”)*;***other obligations/unforeseen evets*** *(“I was obligated to give some sessions that were unplanned”)*;***little time available***	***unfavourable conditions***;***other obligations/unforeseen evets***;***little time available*** *(“I really do not have enough time”)*		***unfavourable conditions***;***other obligations/unforeseen evets***;***little time available***

**Negative consequences**			

**Short-term consequences: *course of action*** *(“I had to work hard and late the day before the meeting”)*; ***well-being*** *(“I was very tense”; “I was worried about not finishing on time”)*; ***private life*** (*“I had to cancel a dinner that night”*)			

**Longer-term consequences: -**	***uncompleted task; concrete implications*** *(“I still do not have foam mats”)*; ***dissatisfaction/remorse*** *(“I think to myself that I’m a little bit stupid [note having done it]”)*		***uncompleted task***; ***concrete implications***; ***dissatisfaction/remorse***

**Neutral/positive consequences**			

**Neutral consequences: *no significant consequence*** *(“There actually weren’t any consequences”)*	**-**		**-**

**Positive consequences: *satisfaction with outcome*** (*“After the meeting, I was relieved that the job had correctly been done”*)	***release of stress/contentment*** *(“The contentment of saying I’ll do it tomorrow”)*; ***time gain*** *(“I had more time to do other things”)*		***release of stress/contentment***; ***time gain***

**Participant 2, male, 32 years, project manager; PPS m = 2.73, PR = 54**			

**Situations a (EO):** daily shopping (Criterion 3 partially fulfilled, Criteria 4–5 not met)	**b (AW):** producing a report for a client (Criteria 4–5 not met)		**c (S):** phoning an old friend (all criteria met)

**Individual causes**			

**Mental/physical state: *fatigue***	**-**		**-**

**Motivation: *general lack of motivation***; ***other preferred actions***	***general lack of motivation***; ***other preferred actions***; ***need for pressure/stimulation seeking***		***general lack of motivation***; ***other preferred actions***

**Emotion/mood: *low mood*** *(“My emotional state, I wasn’t very well”)*	***fear/anxiety*** *(“I had the fear to begin”)*		-

**Thoughts/beliefs: *pretexts/excuses***	high ***self-confidence/self-efficacy***; ***perfectionism*** *(“I must experience a feeling of perfection… that I never have”)*; ***pretexts/excuses***		***perfectionism*** *(“I preferred not to do it, rather than not to do it well”)*; ***pretexts/excuses***

**Skills/knowledge: -**	both low/high ***time-related/organizational skills*** *(“I noticed that there were reasons to wait, there were missing elements. I didn’t want to waste my time working on things that I should have changed later”)*		low ***time-related/organizational skills***

**Autobiographical memory: -**	***knowledge/self-concept/experiences*** (*“It [postponing] happens to me in a lot of situations”)*		***knowledge/self-concept/experiences*** *(“I am not saying that this is the perfect schema, but it’s always worked for me”*)

**Situational causes**			

**Task-related characteristics:** low ***necessity/importance***; ***non-urgency***	***lack of clarity/demandingness*** *(“I was a little bit lost on the objectives”)*; high ***importance*** (*“Because the task was very important”*)		***lack of clarity/demandingness*** *(“Since we met a long time ago, I had the impression that it was more complicated to phone him”)*; ***non-urgency***

**Circumstances/events: *bad weather*** *(“I did not feel like going out. It was cold and raining”)*	**-**		**-**

**Constraints: *other obligations***	***unfavourable conditions***; ***other obligations***		**-**

**Negative consequences**			

**Short-term consequences: -**	***course of action***; ***well-being***; ***private life***		**-**

**Longer-term consequences: *uncompleted task***; ***concrete implications***; ***dissatisfaction*** *(“I was not satisfied [with the situation]”)*	**-**		***uncompleted task***; ***concrete implications***; ***dissatisfaction***

**Neutral/positive consequences**			

**Neutral consequences: -**	**-**		***no significant consequence***

**Positive consequences: *release of stress/contentment***; ***time gain***	***satisfaction with outcome***		**-**

**Participant 3, female, 30 years, administrative assistant; PPS m = 3.73, PR = 90**			

**Situations a (AW):** revising for a continuous assessment test (all criteria met)		**b (EO):** filing a tax return (Criterion 5 partially fulfilled and Criterion 6 not met)	

**Individual causes**			

**Mental/physical state: *fatigue***		***Fatigue***	

**Motivation: *general lack of motivation***; ***need for pressure/stimulation seeking***		***general lack of motivation***	

**Emotion/mood: *fear/anxiety/feeling pressured*** (*“Well, there’s somehow pressure on that”; “I am worried that others will judge me, I’m so afraid of not being able, and I think sometimes it paralyzed me”)*		***anger/irritation***	

**Thoughts/beliefs:** low ***self-confidence/self-efficacy*** (*“If I fail because I didn’t work, I wouldn’t mind, but if I fail while having worked, because I have low self-confidence, I would say that I’m stupid”*); ***pretexts/excuses***		high ***self-confidence/self-efficacy***; ***pretexts/excuses***	

**Skills/knowledge:** *lack of* ***time-related/organizational skills***; ***lack of attention/distractibility*** (*“I started to revise, and then suddenly, I started daydreaming. And the sun was shining. I thought to myself that it would be nice to be outside. And I went to my computer, I opened my emails, I looked at the news…”)*; ***lack of task-related skills*** (*“I gave up when I had difficulties to understand what I did”*)		high ***time-related/organizational skills*** (prioritization); ***lack of attention/distractibility*** *(“I left it [the tax return] on a stack of paper and forgot it”*)	

**Autobiographical memory: *knowledge/self-concept/experiences***		**-**	

**Situational causes**			

**Task-related characteristics: *unattractiveness***		***Unattractiveness***	

**Circumstances/events: -**		**-**	

**Constraints: -**		**-**	

**Negative consequences**			

**Short-term consequences: *well-being*** (*“I was stressed all the time,” “I felt a lot of guilt,” “I lost sleep over it, I was very tired”*)		**-**	

**Longer-term consequences: *incomplete task***; ***dissatisfaction***		***overdue task***	

**Neutral/positive consequences**			

**Neutral consequences: -**		***no significant consequence***	

**Positive consequences: *release of stress/contentment***; ***satisfaction with outcome***		***satisfaction with outcome***	

**Participant 4, female, 27 years, graphic designer; PPS m = 3.64, PR = 87**			

**Situations a (AW):** completing a project for a competition (Criterion 6 not met)	**b (H):** making a doctor’s appointment (Criterion 3 partially fulfilled)		**c (H):** initiating drug therapy (all Criteria met)

**Individual causes**			

**Mental/physical state: -**	**-**		**-**

**Motivation: *generallack of motivation***; ***other preferred actions***	***general lack of motivation***		***general lack of motivation***

**Emotion/mood: *feeling pressured***	**-**		***feeling pressured***

**Thoughts/beliefs: -**	**-**		low ***self-confidence/self-efficacy; perfectionism***; ***pretexts/excuses***

**Skills/knowledge: -**	***lack of attention***		***lack of attention***

**Autobiographical memory: *knowledge/self-concept***	***knowledge/self-concept***		**-**

**Situational causes**			

**Task-related characteristics: *unattractiveness***; ***lack of clarity/demandingness***	***unattractiveness***; ***lack of clarity/demandingness***		***unattractiveness***; ***lack of clarity/demandingness***;***non-urgency***

**Circumstances/events: *holidays*** *(“And after I postponed during three weeks because I leaved in holidays)*	***holidays***		**-**

**Constraints: -**	**-**		**-**

**Negative consequences**			

**Short-term consequences: -**	***well-being***		***well-being***

**Longer-term consequences: -**	***uncompleted task; restricted perspective*** *(“Since I’m being treated, it would have been better if I had seen him [the doctor] before to know if there were alternative treatments depending on the results”)*		***uncompleted task***; ***restricted perspective***

**Neutral/positive consequences**			

**Neutral consequences: -**	***no significantconsequence***		***no significant consequence***

**Positive consequences: *release of stress/contentment; time gain; satisfaction with outcome***	***release of stress***		

**Participant 5, male, 25 years, watchmaker; PPS m = 3.00, PR = 65**			

**Situations a (EO):** opening mail (Criterion 3 to 5 not met)	**b (EO):** storing toilet paper rolls (Criterion 3 partially met and Criteria 5–6 not met)		**c (AW):** calling to receive information about a job (Criterion 3 partially met)

**Individual causes**			

**Mental/physical state: -**	**-**		**-**

**Motivation: -**	**-**		**-**

**Emotion/mood: -**	***anger/irritation***		***fear/anxiety/feeling pressured***

**Thoughts/beliefs: *perfectionism***; ***pretexts/excuses***	**-**		***perfectionism***; ***pretexts/excuses***

**Skills/knowledge:** both low/high ***time-related/organizational skills***	**-**		**-**

**Autobiographical memory: -**	**-**		**-**

**Situational causes**			

**Task-related characteristics:** low ***necessity/importance***; ***non-urgency***	***unattractiveness***; ***lack of clarity/demandingness***;low ***necessity/importance***		***unattractiveness***; ***non-urgency***

**Circumstances/events: -**	**-**		**-**

**Constraints: *otherobligations***; ***little time available***	**-**		**-**

**Negative consequences**			

**Short-term consequences: -**	**-**		***well-being***

**Longer-term consequences: -**	**-**		***uncompleted task***; ***restricted perspective***

**Neutral/positive consequences**			

**Neutral consequences: *no significant consequence***	**-**		**-**

**Positive consequences: -**	***satisfaction with outcome***		***satisfaction with outcome***

**Participant 6, female, 29 years, lawyer; PPS m = 3.36, PR = 80**			

**Situations a (EO):** terminating the lease contract of a parking space (Criterion 3 not met)	**b (AW):** revising for a written examination (Criterion 5 not met)		**c (EO):** booking her scooter in for a service (Criterion 5 not met)

**Individual causes**			

**Mental/physical state: -**	**-**		**-**

**Motivation: *general lack of motivation***	***general lack of motivation***; ***other preferred actions; need for pressure/stimulation seeking***		***general lack of motivation***

**Emotion/mood: -**	**-**		**-**

**Thoughts/beliefs: *pretexts/excuses***	high ***self-confidence/self-efficacy***; ***pretexts/excuses***		***pretexts/excuses***

**Skills/knowledge:** low ***time-related/organizational skills***;***lack of attention/distractibility***; ***lack of task-related skills***	high ***time-related/organizational skills***;***lack of attention/distractibility***		low ***time-related/organizational skills; lack of attention/distractibility***; ***lack of task-related skills***

**Autobiographical memory: *knowledge/self-concept/experiences***	***knowledge/self-concept/experiences***		**-**

**Situational causes**			

**Task-related characteristics: *unattractiveness***; ***lack of clarity/demandingness***; low ***necessity/importance***	***Unattractiveness***		***unattractiveness***; ***lack of clarity/demandingness***

**Circumstances/events: -**	**-**		**-**

**Constraints: -**	**-**		**-**

**Negative consequences**			

**Short-term consequences: -**	***course of action***; ***well-being***		**-**

**Longer-term consequences: *uncompleted task***; ***concrete implications/restricted perspective***; ***dissatisfaction/remorse***	***incomplete task***; ***concrete implications/restricted perspective***; ***dissatisfaction/remorse***		***uncompleted task***; ***concrete implications/restrictedperspective***; ***dissatisfaction/remorse***

**Neutral/positive consequences**			

**Neutral consequences: -**	**-**		**-**

**Positive consequences: *release of stress/contentment; time gain***;***satisfaction with outcome***	***release of stress/contentment; time gain***		**-**


*Note*: PPS m = mean score on the French adaptation of the Pure Procrastination Scale (Rebetez et al., 2014); PR = percentile rank based on the French validation study of the PPS (Rebetez et al., 2014). AW = Academic/Work; EO = Everyday routines/Obligations; S = Social contacts; H = Health.

## General discussion

Comprehensive descriptions of procrastination episodes showed how constituent parts of procrastination definitions were met by six participants from the general population in diverse life domains (academic and work, everyday routines and obligations, health, and social contacts) and provided multifaceted descriptions of their subjective experience. The qualitative analysis of their reports opens new prospects for future research and allows consideration of the current literature from a new perspective, which is discussed according to a dimensional, multifactorial, and integrative approach to procrastination.

### Towards a dimensional approach to procrastination

Interestingly, only three participants, in one situation each, met all criteria regarding the constituent parts of procrastination definitions. In addition, the criteria that were met differed across participants and situations. This calls into question the categorical approach to procrastination, which focuses on the presence or absence of certain criteria (e.g., procrastination is present when a specific number of criteria are identified and absent when these criteria are not met), and establishes a boundary between “normal” and “pathological” procrastination. Indeed, despite the lack of consensus on the definitions of procrastination, it is striking that most authors make a distinction between normal and pathological functioning. Sabini and Silver (1982) have thus described procrastination as the “psychopathology of everyday life.” In fact, most attempts at establishing a clear definition of procrastination involve a categorical approach, the presence of procrastination being defined, for example, as the fulfilment of seven criteria (see Klingsieck, 2013a). However, this categorical conception is confronted with the problem of “subthreshold symptomatology” (e.g., a person who meets six of the seven criteria of procrastination would not be considered as someone who procrastinates), which is often accompanied by concrete complaints and/or significant psychological distress (see Bentall, 2003, for a critical analysis of the categorical approach).

**Figure 3:**
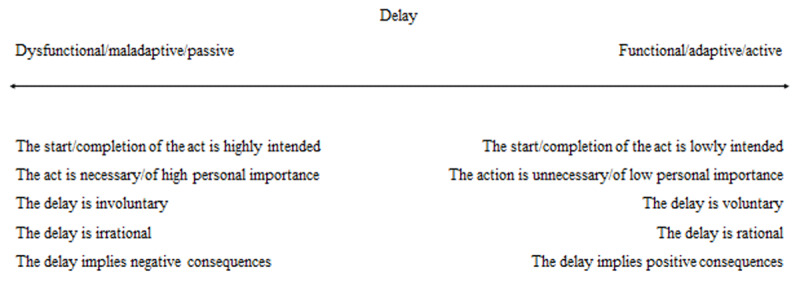
A dimensional approach to procrastination.

By contrast, a dimensional approach considers that psychological difficulties are dimensional in nature, falling along a continuum (Haslam, Holland, & Kuppens, 2012). From this perspective, the main constituent parts of procrastination definitions provided in the literature (e.g., the intentionality of the action and the irrationality of the delay) are continuous (see ***Figure 3***) rather than dichotomous (“present/absent”). The associations of multiple components on one end of the continuum could lead to “dysfunctional/maladaptive/passive” delay (e.g., Beswick & Mann, 1994; Ferrari et al., 1995; Silver & Sabini, 1981), whereas procrastination could be “functional/adaptive/active” when multiple components are located at the other end of the continuum (e.g., Chu & Choi, 2005; Ferrari, 1993; McCown & Roberts, 1994). In most cases, however, the associations between different components of procrastination are varied, with some components seeming to fall at one end of the continuum (e.g., the delay is voluntary and irrational), and other aspects seeming to be located at the other end (e.g., there is a low intent to start/complete the act and the delay implies positive consequences) or to fall somewhere in between (e.g., the act is necessary but not really important). Adopting a dimensional perspective allows one to understand what determines the variability of procrastination manifestations, rather than focusing on what distinguishes procrastination from other forms of delay.

However, qualitatively different psychological processes could also be involved in adaptive versus maladaptive procrastination, suggesting that there are possibly more than one continuum, such as one for active – adaptive procrastination and one for passive – maladaptive procrastination, which do not necessarily involve the same underlying psychological processes (e.g., low to high prioritization capacity for adaptive procrastination and poor to optimal self-regulatory processes for maladaptive procrastination). In fact, one could more precisely hypothesize that these two continuums are orthogonal, thus raising the possibility that maladaptive procrastination results from dysfunctional processes on both continuums simultaneously. Participant 3 may illustrate this hypothesis inasmuch as she showed the greatest level of general procrastination and reported individual causes, especially emotional and self-related causes, as well as motivational and self-regulation-related difficulties, with few aspects of active procrastination (e.g., prioritization) at least for one reported episode of procrastination. Further studies should however more specifically explore the continuum hypothesis in procrastination.

### Towards a multifactorial and integrative approach to procrastination

The descriptions of procrastination episodes reported by different participants in various situations yielded a category system containing a broad range of causes (individual and situational) of procrastination. Many of them have been extensively discussed in the literature, even if they sometimes led to contrasting interpretations, such as themes relating to motivation (Chu & Choi, 2005; Ferrari, 1992; Lee, 2005), emotion/mood (Sirois & Pychyl, 2013), thoughts/beliefs (Haycock et al., 1998; Pychyl & Flett, 2012; Steel, 2007), skills/knowledge (Choi & Moran; Harriott et al., 1996; Steel, 2007), and task characteristics (Blunt & Pychyl, 2000). The descriptions further yielded another category system containing negative and neutral/positive consequences of procrastination, most of which have also been discussed in previous studies (Chu & Choi, 2005; Schraw et al., 2007; Sirois & Pychyl 2013). Interestingly, however, participants’ descriptions also point to additional causes and consequences that have not gained much attention in previous research, in particular the context of procrastination (e.g., *fatigue* or *bad weather*) and conditions hindering/slowing down the course of action (e.g., *other obligations/unforeseen events, little time available*). Surprisingly, some participants also expressed high self-confidence and self-efficacy to justify the delay of a decision or an action. Several participants also mentioned the absence of significant consequences for their procrastination behaviours. These data thus underline the high diversity of procrastination causes and consequences (including its context/external reasons, some functional aspects, or the absence of consequence).

Another observation was that the descriptions showed differences among participants regarding causes and consequences of procrastination, which may be linked to different profiles of procrastination (see Rebetez et al., 2015). For example, Participant 5 (one of the participants with the lowest score of procrastination) seems to deliberately decide to delay tasks, mainly because of external constraints (e.g., *other obligations, little time available*) and the way he prioritizes his actions; together with the few negative consequences reported and his satisfaction with the outcome, this profile could be linked to active procrastination (see Chu & Choi, 2005). By contrast, Participant 3 (with the highest score of procrastination) expressed the most individual causes overall (in particular emotional and self-related causes, as well as motivational and self-regulation-related difficulties) and numerous consequences (including negative consequence during the process of procrastination, in particular for well-being, and afterward); this profile could be linked to a previously established emotional profile (where procrastination could be viewed as a way to immediately regulate mood at the expense of long-term goals) or a previously established unregulated profile (where procrastination could be viewed as a reflection of a larger self-regulatory failure) (see Rebetez et al., 2015). In addition, within participants, procrastination manifested differently in terms of its causes and consequences, depending on the situation, supporting a domain-specific view of procrastination (Klingsieck, 2013b; see also Subotnik, Steiner, & Chakraborty, 1999). For example, although Participant 3 expressed the most individual causes overall, one situation (work related) was related to dysfunctional aspects of procrastination, whereas the other situation (obligations/routines) was related to adaptive aspects (prioritization). These data thus underline the high variability of procrastination manifestations across individuals and situations, although all participants were relatively comparable in terms of their general tendency to procrastinate and, together with data underlining the high diversity of its causes and consequences, call for a multifactorial and integrative approach to this phenomenon.

In sum, the interviews illustrate that different aspects of delay, both dysfunctional and functional, seem to lie one continuum or possibly two orthogonal continuums and vary between individuals and across situations. In most cases, the associations between the constituent parts of procrastination (according to common definitions in the literature) were highly heterogeneous, with some components seeming to lie at one end of the continuum (dysfunctional aspects), other components at the other end (functional aspects) of the continuum or on another (orthogonal) continuum, and still others somewhere in between whether on one or two orthogonal continuums.

In addition, rich and multifaceted descriptions of procrastination episodes were obtained, showing a variety of individual/situational causes and consequences, which revealed the implication of variables that have been little explored so far, such as the context of procrastination, conditions hindering/slowing down the course of action, high self-confidence and self-efficacy, and absence of significant consequences. Moreover, the descriptions showed a high variability of procrastination manifestations across individuals and situations, with more or less dysfunctional or functional aspects. Overall, these data stress the need for a dimensional, multifactorial, and integrative approach to procrastination.

From this perspective, a general theoretical model such as the mediating psychological processes model (Kinderman, 2005, 2009, 2014; Kinderman & Tai, 2007) could be applied to unify the different theoretical views of procrastination and to conceive procrastination from a dimensional perspective and capture its multifaceted and dynamic nature. This model postulates that biological, social, and circumstantial (situational) factors can lead to mental health problems through their conjoint effects in influencing or disrupting psychological processes (Kinderman, 2005). Following this view, biological (e.g., genes, sex, age), social (e.g., gender identity, parenting style, education, nationality), and situational (e.g., characteristics of the task, domain, context) factors could contribute to the onset and maintenance of procrastination through their conjoint effects on different psychological processes (e.g., by modifying the way individuals control their impulsions and thoughts, engage in goal pursuit, regulate their emotions, or integrate experiences according to their values, beliefs, personal standards and expectancies), which may lead to both negative and positive consequences.

A number of limitations on the current qualitative study should be considered. First, the study does not allow for generalizing the results, which apply specifically to the interviewed participants. Second, the sample is small (although it still demonstrates the great variability of procrastination and manifestations) and the life domains represented are probably non-exhaustive (although they still cover the main domains described in the literature; see for example Klingsieck, 2013b). Third, it was based on individual experiences, which are bound to be subjective and biased (e.g. memory bias, attributional bias, social desirability bias). In particular, descriptions of procrastination episodes probably vary according to the time they occurred (e.g. for longer interval, more general semantic descriptions could be expected). Fourth, even though a purposely-broad definition of procrastination (i.e. “putting [something] off until a later time”) was given to the participants at the beginning of the interview, it implies that any form of delay (either procrastination, prioritization, delays ascribed to circumstances or other kinds of delay) was eligible as material for the present qualitative analysis. This precludes from drawing a firm conclusion regarding a positive end of the procrastination-continuum or a positive form of procrastination, as these positive evidenced features might also characterize related behaviours (e.g., prioritization). However, even if procrastination is considered a self-regulatory failure (as the prevailing literature in the field appears to demonstrate), our results suggest that, at least, individuals with a relatively high general tendency to procrastinate (as measured by the Pure Procrastination Scale) felt that some of their dilatory behaviours entail positive aspects. Finally, although this study aimed to examine procrastination from a dimensional perspective, participants have been included only if they reported a medium to elevated score of general procrastination on the Pure Procrastination Scale. This inclusion criterion may have led to an overestimation of the factors involved in maladaptive procrastination at the expanse of factors involved in adaptive procrastination. Consequently, further studies adopting a dimensional perspective should include participants with the whole range of scores on the Pure Procrastination Scale.

In conclusion, and despite these limitations, this work provides new insight into the understanding of procrastination in everyday life within the general population. The diversity of the manifestations of procrastination behaviours call for individualized evaluations, which aim at identifying the specific psychological processes that are responsible for the onset and maintenance of procrastination for a particular person in a given situation.

This work was part of the first author’s PhD thesis under the supervision of Professor Martial Van der Linden. In his research, Professor Van der Linden made a point of supporting the need to adopt a dimensional, multifactorial and integrative approach toward psychopathological states by promoting the identification of the various psychological processes involved in problematic behaviours, underlining interindividual differences and better characterising the interactions between psychological processes and other variables at the biological and environmental levels. Such an approach might definitely help clinicians in the appraisal of the complexity of potential problematic behaviours such as procrastination and promote targeted and more effective therapy in clinical settings.
